# Effect of Visual Crowding on Quality of Life in Glaucoma Patients

**DOI:** 10.1167/iovs.67.12.13

**Published:** 2026-10-07

**Authors:** Marcus Guerreiro-Filho, Davina A. Malek, Ali Azizi, Ivan M. Tavares, Felipe A. Medeiros

**Affiliations:** 1 Bascom Palmer Eye Institute, University of Miami, Miami, Florida, United States; 2 Department of Ophthalmology and Visual Sciences, Paulista School of Medicine, Universidade Federal de São Paulo, São Paulo, Brazil

**Keywords:** glaucoma, open-angle, visual crowding, quality of life, visual fields

## Abstract

**Purpose:**

To investigate the association between visual crowding and vision-related quality of life (QoL) in patients with glaucoma.

**Methods:**

This cross-sectional study included 50 patients diagnosed with POAG. Visual crowding was quantified by measuring the critical spacing (S_critical_). The S_critical_ was assessed in each visual field quadrant. For each eye, a global S_critical_ was calculated as the mean of the eligible quadrant-specific values. Vision-related QoL was assessed using the 25-item National Eye Institute Visual Function Questionnaire (NEI VFQ-25), scored using Rasch analysis. Associations between the S_critical_ and the NEI VFQ-25 Rasch-calibrated scores were examined using generalized estimating equations, with multivariable models adjusted for age and standard automated perimetry 24-2 mean deviation.

**Results:**

Among 50 patients with POAG (93 eyes; mean age, 67.9 ± 9.2 years), the mean Rasch-calibrated NEI VFQ-25 score was 59.9 ± 18.8. In univariable analyses, each 1-degree increase in the better-eye global S_critical_ was associated with a 5.69-point lower NEI VFQ-25 score (95% confidence interval, −10.43 to −0.96; *P* = 0.018), and each 1-degree increase in the worse-eye S_critical_ was associated with a 2.23-point lower score (95% confidence interval, −4.38 to −0.08; *P* = 0.042). In multivariable analysis adjusting for age and standard automated perimetry mean deviation, the better-eye S_critical_ remained independently associated with a worse QoL (−5.02 points per degree; 95% confidence interval, −9.59 to −0.45; *P* = 0.031).

**Conclusions:**

Visual crowding is independently associated with worse vision-related QoL in patients with glaucoma, even after adjustment for standard perimetric loss. These findings suggest that visual crowding captures clinically meaningful aspects of visual dysfunction beyond conventional perimetry.

Visual crowding refers to the impaired ability to recognize an object when it is surrounded by nearby visual clutter, even though that same object can be readily identified when presented in isolation. This phenomenon represents a fundamental limitation of visual perception, particularly in peripheral vision, and plays a critical role in everyday activities that require object recognition in complex environments, such as reading, driving, visual search, and navigating through scenes.^1^^,^^2^ Importantly, crowding does not primarily reflect a loss of visual acuity or contrast sensitivity; rather, it reflects a failure of feature integration, whereby information from adjacent objects is inappropriately pooled, leading to perceptual jamming or confusion.

Historically, visual crowding was first described in the peripheral vision of individuals with normal sight and in the central vision of patients with amblyopia. More recently, compelling evidence has demonstrated that crowding is also abnormally increased in eyes with glaucoma, including individuals with preperimetric glaucoma, whose visual field retains relatively preserved sensitivity on standard automated perimetry (SAP).^3^^–^^6^ These findings suggest that glaucomatous damage affects visual processing in ways that extend beyond conventional measures of visual field loss and may contribute substantially to the real-world visual difficulties experienced by patients.

Indeed, there is growing recognition that glaucoma impairs multiple aspects of visual function not captured by visual acuity or SAP alone. Prior studies have documented impairments in visual object categorization under reduced-contrast conditions,^7^ as well as abnormalities in visual exploration strategies and saccadic eye movements,^8^ which in turn are associated with poorer performance on visually demanding tasks such as reading,^9^ face recognition,^10^ searching for objects in complex scenes,^11^ and driving.^12^ In this context, crowding provides a particularly compelling framework for understanding functional impairment in glaucoma, as it reflects limitations in object recognition under cluttered conditions, which are precisely the circumstances encountered in daily life.

Supporting this notion, Ogata et al.^3^ demonstrated that patients with glaucoma show significantly increased critical spacing (S_critical_) for crowding compared with healthy controls, even at early stages of the disease. Subsequently, Stievenard et al.^5^ reported that excessive sensitivity to central crowding may underlie the difficulties with reading and face perception experienced by some patients with glaucoma. Together, these findings suggest that abnormal crowding represents a clinically meaningful source of visual disability and may contribute to vision-related quality of life (QoL) impairment in glaucoma. Such impacts are traditionally assessed using patient-reported outcome measures, including instruments such as the 25-item National Eye Institute Visual Function Questionnaire (NEI VFQ-25),^13^ yet the specific contribution of visual crowding to patient-reported visual disability has not been directly evaluated.

In the present study, we assessed visual crowding in individuals with POAG and investigated its association with vision-related QoL as measured by the NEI VFQ-25 questionnaire. By linking a psychophysically defined measure of crowding to subjective visual disability, this study aimed to clarify whether visual crowding represents a key functional pathway through which glaucomatous damage affects the everyday visual experience of patients.

## Methods

Participants were selected from an ongoing prospective longitudinal investigation designed to assess functional impairment in glaucoma. Data collection was conducted at the Visual Performance Laboratory at Duke University, and data analysis was performed at the Bascom Palmer Eye Institute of the University of Miami. The institutional review boards of both institutions approved the study methods, and written informed consent was obtained from all participants. The study adhered to the laws of the Health Insurance Portability and Accountability Act, and all procedures complied with the tenets of the Declaration of Helsinki.

All participants underwent a comprehensive ophthalmologic examination, including a review of medical history, visual acuity assessment using an Early Treatment Diabetic Retinopathy Study chart (expressed as the logMAR for statistical analyses), slit-lamp biomicroscopy, IOP measurement using Goldmann applanation tonometry, corneal pachymetry, gonioscopy, and dilated fundus examination with a 78-diopter lens. Structural and functional assessments included stereoscopic optic disc photography, SAP using the 24-2 Swedish Interactive Threshold Algorithm Standard on the Humphrey Field Analyzer II (model 750; Carl Zeiss Meditec, Inc., Dublin, CA, USA), and spectral-domain optical coherence tomography (SD-OCT) using the Spectralis SD-OCT system (Heidelberg Engineering, Heidelberg, Germany). Global peripapillary retinal nerve fiber layer (RNFL) thickness was measured using a 3.45-mm circular scan centered on the optic disc. All OCT images were reviewed for adequate quality, with a signal strength of greater than 15 dB, before inclusion in the analyses. Only individuals with open angles on gonioscopy were included. Patients with coexisting retinal disease, uveitis, or nonglaucomatous optic disc neuropathy were excluded.

Glaucoma was defined by the presence of two or more consecutive abnormal SAP examinations at baseline, characterized by a pattern standard deviation with a *P* value of less than 0.05 and/or glaucoma hemifield test results outside normal limits, together with evidence of glaucomatous optic neuropathy based on masked assessment of stereoscopic optic disc photographs. This assessment was done by the referring provider and confirmed by a fellowship-trained glaucoma specialist. Only reliable SAP tests were considered, defined as those with fewer than 33% fixation losses and fewer than 15% false-positive responses, consistent with established reliability criteria in glaucoma research.^14^^–^^17^ Visual fields were reviewed by two glaucoma specialists (MGF and IMT), with disagreements resolved by consensus with the senior author (FAM) and excluded when the results were affected by artifacts or nonglaucomatous factors, including eyelid or lens rim artifacts, fatigue effects, inattention, inappropriate fixation, or abnormalities suggestive of visual pathway disease other than glaucoma. Patients were eligible if they had a diagnosis of glaucoma in at least one eye. In an exploratory analysis to further characterize the relationship between glaucoma severity and visual crowding, eyes were classified according to SAP 24-2 mean deviation (MD) as having mild (MD ≥ −6 dB), moderate (−12 dB ≤ MD < −6 dB), or severe (MD < −12 dB) glaucoma.

In addition to clinical testing, participants completed socioeconomic questionnaires that collected information on demographics, ocular and medical history, marital status, health insurance coverage, educational attainment, and income.

### Visual Crowding Assessment

The crowding measurement protocol used in this study was adapted from a previously published psychophysical paradigm developed by our group,^3^ with minor modifications designed to improve testing efficiency and reduce examination time while preserving the fundamental principles of the original methodology.

Visual crowding was assessed by quantifying the S_critical_, defined as the minimum center-to-center distance between a target and its flanking elements at which target recognition performance is degraded. Testing was conducted using a 27-inch LCD monitor in a darkened room, with the screen centered at the participant's eye level and viewed at a distance of 40 cm. Crowding testing was performed on the same day as clinical examinations, following a standardized rest period after the completion of clinical testing. The order of testing was identical across participants and study visits.

Each visual field quadrant was assessed in a separate block during the same visit, with four blocks attempted per eye. For each block, a fixation target (“+”) was positioned at one corner of the screen (Fig. 1), such that the target stimulus appeared at 10° of eccentricity diagonally opposite the fixation point, at polar angles of 45°, 135°, 225°, and 315°. This configuration allowed the independent assessment of crowding in the superior temporal, superior nasal, inferior temporal, and inferior nasal quadrants. The target stimulus consisted of a randomly selected Sloan letter (D, H, K, N, O, R, S, V, or Z), flanked radially by two Sloan letters presented at variable distances from the target. All stimuli were rendered in black on a white background.

**Figure 1.**
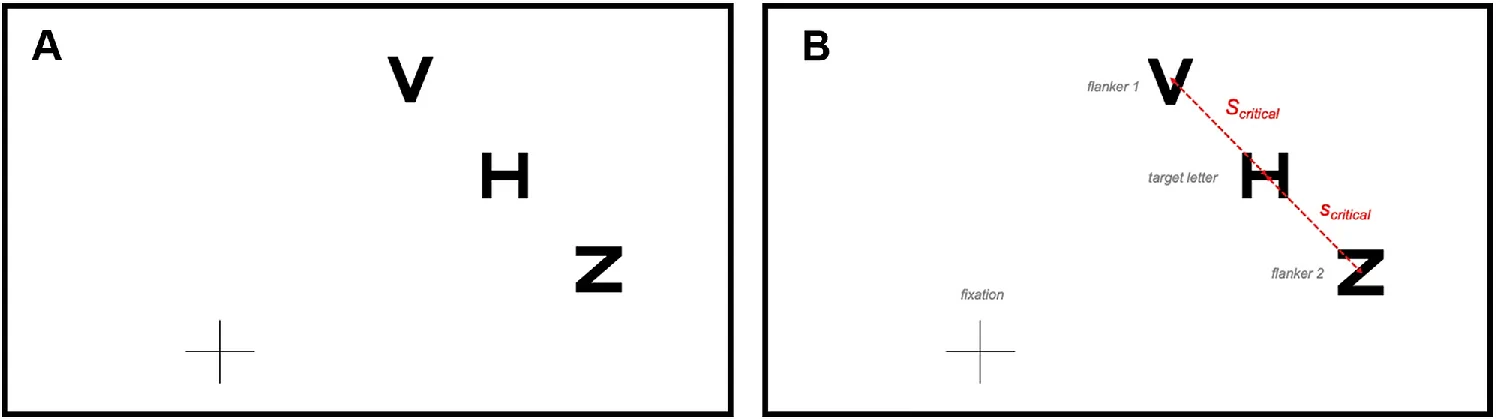
(**A**) The visual crowding test for the superior temporal quadrant for the right eye or superior nasal quadrant for the left eye. Subjects were instructed to fixate at the cross (+) and call out the letter in the center (in this example, H). Each eye and quadrant was tested separately. (**B**) The minimum distance (in degrees) from the center of the target letter and the center of the flankers (V and Z) for which the subject was able to discriminate the target letter correctly was defined as the S_critical_.

Participants first completed three practice trials to become familiar with the task. During testing, they were instructed to fixate on the corner fixation target before stimulus onset and to maintain this fixation throughout each trial. Stimuli were presented briefly, and participants were asked to verbally identify the central target letter located between the two flankers. The examiner repeated the response aloud and recorded it using a keyboard. No feedback regarding response accuracy was provided.

Before the initiation of the crowding measurement, an initial calibration phase was performed for each quadrant to ensure that participants could reliably identify the target stimulus. Fixed-size target and flanker letters subtending 1.3° of visual angle were initially presented. The letter size was then adaptively adjusted by the software, while maintaining a fixed target–flanker spacing, until the smallest letter size that could be correctly identified twice was determined for each participant. Quadrants in which participants were unable to meet this calibration criterion did not proceed to the S_critical_ estimation and were not included in the subsequent crowding analyses. This procedure helped to ensure adequate target visibility and minimize the confounding effects of localized scotomas. The visibility of the flanking letters was not independently assessed. Notably, letter size itself is not expected to influence crowding magnitude; whereas visual acuity depends on object size, crowding is governed by center-to-center spacing, with its spatial extent scaling with retinal eccentricity.^18^

Following this calibration step, S_critical_ estimation was performed using the QUEST Bayesian adaptive psychophysical procedure. QUEST was initialized using a fixed prior structure. The nominal prior was centered at log_10_(0.3°) = −0.52, corresponding with a fixed starting target–flanker spacing of 0.3°. The QUEST prior was broad, with a fixed prior standard deviation of 2 log_10_ units. The nominal QUEST search domain was ±3 log_10_ units around the fixed prior center, corresponding with a range of 6 in log_10_ spacing units. These implementation parameters were identical across participants and quadrants and were not calculated from either the calibrated letter size or the fixed target–flanker spacing used during calibration. Rather than testing predetermined target–flanker distances, QUEST adaptively selected center-to-center target–flanker spacing on a trial-by-trial basis according to the participant's cumulative response history. Observers completed a nine-alternative forced-choice letter identification task using the nine Sloan letters listed elsewhere in this article, corresponding with a theoretical chance performance level of 11.1%.

To maintain proportionality between the stimulus size and spacing and prevent physical overlap between the target and flanking letters, the target letter size was dynamically scaled to the target–flanker spacing selected by QUEST using a fixed 1.0:1.2 size-to-spacing ratio throughout the testing. Thus, target–flanker spacing, rather than letter size, was the adaptive stimulus variable used for S_critical_ estimation. This approach minimized the influence of physical masking while preserving the spatial characteristics of the crowding stimulus.

Target and flanking letters were presented for 200 ms in each trial. Each quadrant-specific block consisted of 20 trials, resulting in a total of 80 trials per eye. The entire crowding assessment required approximately 20 minutes per eye, including the calibration and practice trials. Short breaks were allowed between quadrant blocks upon participant request, and a standardized 3-minute break was provided between eyes to minimize fatigue.

Crowding was defined as a reduction in target identification accuracy when target–flanker spacing fell below a critical value. During the 20-trial sequence for each eligible quadrant, QUEST adaptively selected target–flanker spacings based on the participant's cumulative pattern of correct and incorrect responses. After completion of the 20 trials, the target–flanker spacings sampled by QUEST and the corresponding correct and incorrect responses were used to fit a logistic psychometric function relating target identification accuracy to target–flanker spacing. For each quadrant, the S_critical_ was defined as the spacing corresponding to 75% correct performance. For each eye, a global S_critical_ value was calculated as the mean of all eligible quadrant-specific measurements. To further evaluate the reliability of the 20-trial QUEST-like procedure, we performed a post hoc Monte Carlo robustness simulation using the task parameters and implementation-informed assumptions described elsewhere in this article, with full details provided in the Supplementary Appendix and Supplementary Code S1.

Because the severity of glaucomatous damage tends to differ between the two eyes, the worse and better eyes were determined based on the MD value from the SAP 24-2 test, where a more negative MD was considered to be more severe glaucoma.

### NEI VFQ-25 Questionnaire

Patient-reported QoL was evaluated using the NEI VFQ-25 within 1 month of the visual crowding test. This questionnaire consists of 25 questions designed to assess the dimensions of self-reported vision-targeted health status that are relevant for patients with chronic eye diseases.^19^ The NEI VFQ-25 responses were calibrated using a Rasch analysis to obtain the final estimates of person measures or Rasch scores, as previously described in detail.^20^ Briefly, an Andrich rating scale model was used to estimate item difficulty and person ability measures on a logit scale. Consistent with prior Rasch evaluations demonstrating the multidimensional structure of the NEI VFQ-25, items from the dependency, mental health, and role limitation subscales were excluded because they represented a separate socioemotional dimension, whereas ocular pain items were excluded because they did not adequately fit the Rasch model. Accordingly, the final Rasch-calibrated visual functioning score included items from the general vision, near vision, distance vision, peripheral vision, color vision, and social functioning subscales. Person ability scores were rescaled linearly to range from 0 to 100 with higher scores indicating better vision-related QoL. In this study, the Rasch scores were used to express the location of each respondent on a linear scale representing the degree of impairment as measured by the NEI VFQ-25 and were used for subsequent statistical analyses.^21^

### Statistical Analyses

Descriptive statistics were used to summarize the study variables, with means and standard deviations reported for normally distributed data and medians with interquartile ranges (IQRs) for non-normally distributed data. Normality assumptions were assessed by visual inspection of histograms and formally tested using the Shapiro–Wilk test.

Univariable regression analyses were initially performed to examine associations between vision-related QoL, as measured by the NEI VFQ-25 score, and the visual crowding S_critical_, SAP measures, and demographic characteristics, to identify potential confounders associated with lower NEI VFQ-25 scores. Multivariable regression models were then constructed to evaluate the independent association between visual crowding and the NEI VFQ-25 score, adjusting for age and the severity of glaucomatous damage, as quantified by the SAP 24-2 MD.

Generalized estimating equations with robust sandwich variance estimators were used to account for within-subject correlation arising from the measurements obtained from both eyes of the same individual. Analyses incorporating the global RNFL thickness, measured by SD-OCT, as an additional covariate were also conducted in the multivariable models. Differences in the eligible S_critical_ across the four visual field quadrants were assessed across all eyes using a Wald χ^2^ test within a linear mixed-effects model with random effects for patient and eye, with post hoc pairwise comparisons adjusted using the Bonferroni correction. In a sensitivity analysis, the multivariable models were repeated using the worst-performing quadrant of each eye, defined as the quadrant with the highest S_critical_, in place of the global mean S_critical_. Agreement between the average S_critical_ and the worst-quadrant S_critical_ was assessed using the Pearson correlation coefficient. All statistical analyses were performed using commercially available software (Stata, version 18; StataCorp LP, College Station, TX, USA). A two-sided significance level (type I error) of 0.05 was adopted for all analyses.

## Results

This study included 93 eyes from 50 patients with POAG. The mean baseline patient age was 67.9 ± 9.2 years; 23 patients (46%) were female and 15 (30%) self-identified as Black or African American. The mean Rasch-calibrated NEI VFQ-25 score was 59.9 ± 18.8. Demographic and clinical characteristics are summarized in Table 1. Based on the SAP 24-2 MD, better eyes had a median MD of −1.75 dB (IQR, −4.34 to −0.54), whereas worse eyes had a median MD of −5.93 dB (IQR, −11.06 to −1.51 dB). The mean S_critical_ was 4.3 ± 1.5 degrees in better eyes and 4.7 ± 2.1 degrees in worse eyes.

**Table 1.** Demographics and Clinical Characteristics of Subjects Included in the Study

Characteristic	
Age, years	67.9 ± 9.2
Sex, female	46%
Race, Black or African American	30%
NEI VFQ-25 Rasch-calibrated score	59.9 ± 18.8
Worse eye SAP 24-2 MD, dB	−5.93 (−11.06 to −1.51)
Mean ± standard deviation	−7.18 ± 6.7
Better eye SAP 24-2 MD, dB	−1.75 (−4.34 to −0.54)
Mean ± standard deviation	−3.05 ± 4.2
Worse eye S_critical_, degree	4.7 ± 2.1
Better eye S_critical_, degree	4.3 ± 1.5
Glaucoma severity (eyes), *n* (%)	
Mild	63 (67.7)
Moderate	17 (18.3)
Severe	13 (14.0)

Values are reported as mean ± standard deviation, percentage, or *n* (%), as appropriate. SAP MD values are reported as median (IQR) and mean ± standard deviation.

Of the 372 expected quadrant measurements from 93 study eyes, 24 quadrants (6.5%) from 12 eyes of 9 patients did not meet the calibration criterion and were not included in the S_critical_ estimation. These 12 eyes included 7 eyes with mild, 3 with moderate, and 2 with severe glaucoma. The remaining 348 eligible quadrant measurements (93.5%) were included in the subsequent crowding analyses.

In the univariable analyses, both the SAP MD and visual crowding were associated with worse vision-related QoL. Lower SAP 24-2 MD was significantly associated with lower Rasch-calibrated NEI VFQ-25 scores in both the worse eye (−0.81 points per 1 dB lower MD; 95% confidence interval [CI], −1.56 to −0.05; *P* = 0.035) and the better eye (−0.94 points per 1 dB lower MD; 95% CI, −1.65 to −0.24; *P* = 0.008). A greater S_critical_ was also significantly associated with lower Rasch-calibrated NEI VFQ-25 scores in both eyes, with a stronger effect observed for the better eye. Specifically, each 1-degree increase in the S_critical_ was associated with a 2.23-point lower Rasch-calibrated NEI VFQ-25 score for the worse eye (95% CI, −4.38 to −0.08; *P* = 0.042) and a 5.69-point lower score for the better eye (95% CI, −10.43 to −0.96; *P* = 0.018). Age, sex, and race were not significantly associated with the Rasch-calibrated NEI VFQ-25 scores in univariable models (Table 2). Figure 2 illustrates the inverse relationship between the S_critical_ and patient-reported QoL for both the better and the worse eyes.

**Table 2.** Univariable Analyses of the Relationships Between Rasch Scores From the NEI VFQ-25 and Predictor Variables

	Univariable Model	
Characteristic	Effect on NEI VFQ-25 (Rasch score) Coefficient (95% CI)	*P* Value
Age, per decade older	1.39 (−3.31 to 6.10)	0.561
Sex, female	4.72 (−4.93 to 14.39)	0.338
Race, Black or African American	6.16 (−3.56 to 15.89)	0.214
Worse eye SAP 24-2 MD, per dB lower	−0.81 (−1.56 to −0.05)	**0.035**
Better eye SAP 24-2 MD, per dB lower	−0.94 (−1.65 to −0.24)	**0.008**
Worse eye S_critical_, per degree greater	−2.23 (−4.38 to −0.08)	**0.042**
Better eye S_critical_, per degree greater	−5.69 (−10.43 to −0.96)	**0.018**

Boldface indicates statistical significance (*P* < 0.05).

**Figure 2.**
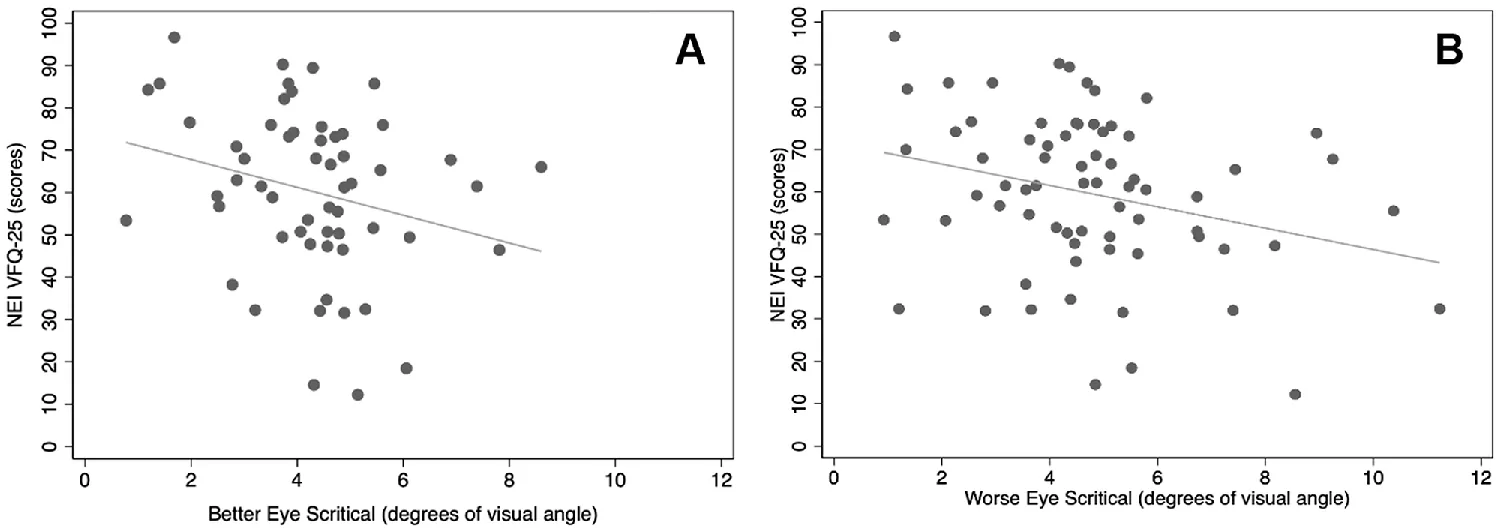
(**A**) Scatterplot of the relationship between the better eye global S_critical_ and the NEI VFQ-25 Rasch scores. (**B**) Scatterplot of the relationship between the worse eye global S_critical_ and the NEI VFQ-25 scores.

To assess whether the association between crowding and QoL persisted after accounting for glaucomatous visual field loss, multivariable models were constructed adjusting for age and the SAP 24-2 MD. In these analyses, the greater better-eye S_critical_ remained significantly associated with lower Rasch-calibrated NEI VFQ-25 scores (−5.02 points per 1-degree increase; 95% CI, −9.59 to −0.45; *P* = 0.031). The better-eye MD also remained independently associated with worse QoL in this model (−0.68 points per 1-dB lower MD; 95% CI, −1.30 to −0.05; *P* = 0.033). In contrast, the worse-eye S_critical_ was no longer significantly associated with the Rasch-calibrated NEI VFQ-25 score after adjustment (−1.66 points per 1-degree increase; 95% CI, −3.80 to 0.48; *P* = 0.129), and the worse-eye MD was also not statistically significant in that model (*P* = 0.269) (Table 3).

**Table 3.** Multivariable Models Assessing the Effect of Crowding Critical Spacing (S_critical_) in the Better and Worse Eyes on Rasch Scores From the NEI VFQ-25

	Multivariable Model Better Eye		Multivariable Model Worse Eye	
	Effect on NEI VFQ-25		Effect on NEI VFQ-25	
	(Rasch score)		(Rasch score)	
Characteristic	Coefficient (95% CI)	*P* Value	Coefficient (95% CI)	*P* Value
Age, per decade older	3.51 (−1.07 to 8.11)	0.133	−1.09 (−6.20 to 4.01)	0.675
S_critical_, per degree greater	−5.02 (−9.59 to −0.45)	**0.031**	−1.66 (−3.80 to 0.48)	0.129
SAP 24-2 MD, per dB lower	−0.68 (−1.30 to −0.05)	**0.033**	−0.45 (−1.25 to 0.34)	0.269

Boldface indicates statistical significance (*P* < 0.05).

We also evaluated models including RNFL thickness. In the better-eye model, a greater S_critical_ remained independently associated with lower Rasch-calibrated NEI VFQ-25 scores (−5.19 points per 1-degree increase; 95% CI, −9.74 to −0.63; *P* = 0.025), and the lower SAP 24-2 MD also remained significantly associated with a worse QoL (−0.78 points per 1-dB lower MD; 95% CI, −1.56 to −0.01; *P* = 0.048). In contrast, RNFL thickness was not independently associated with QoL (1.03 points per 10-µm lower RNFL thickness; 95% CI, −2.78 to 4.85; *P* = 0.594). Similar findings were observed in the worse-eye model, in which neither S_critical_, SAP 24-2 MD, nor RNFL thickness was significantly associated with QoL (Supplementary Table S1).

Among the 93 eyes, 63 (67.7%) were classified as having mild glaucoma, 17 (18.3%) as having moderate glaucoma, and 13 (14.0%) as having severe glaucoma (Table 1). The mean S_critical_ increased progressively with disease severity, measuring 4.14 ± 0.19, 4.89 ± 0.48, and 6.42 ± 0.64 degrees in the mild, moderate, and severe groups, respectively. In this analysis, glaucoma severity was significantly associated with a greater S_critical_ (overall *P* = 0.0019).

The S_critical_ differed modestly across quadrants (overall χ^2^ = 9.54; *P* = 0.023). The mean S_critical_ values were 5.02, 4.17, 4.82, and 4.59 degrees for the superior temporal, superior nasal, inferior nasal, and inferior temporal quadrants, respectively. Post hoc pairwise comparisons with Bonferroni correction showed that this difference was primarily driven by a lower S_critical_ in the superior nasal quadrant compared with the superior temporal quadrant (*P* = 0.020), whereas no other pairwise comparisons were statistically significant. We then performed additional analyses using the worst-performing quadrant of each eye rather than the average S_critical_ value. In the better-eye model, the greater worst-quadrant S_critical_ remained significantly associated with lower Rasch-calibrated NEI VFQ-25 scores after adjustment for age and SAP 24-2 MD (−5.31 points per 1-degree increase; 95% CI, −9.40 to −1.21; *P* = 0.011). The correlation between the average S_critical_ and worst-quadrant S_critical_ was high (*r* = 0.89), indicating that the primary findings were robust to the method used to summarize crowding across the quadrants (Supplementary Table S2).

## Discussion

In this study, a greater global S_critical_ in the better eye was associated with a worse QoL in patients with glaucoma. This association remained significant after adjustment for age and visual field loss, as measured by the MD, suggesting that visual crowding captures aspects of visual dysfunction relevant to QoL that are not fully reflected by standard perimetry alone. These findings support the potential value of visual crowding assessment as a complementary measure for understanding patient-reported functional impairment in glaucoma.

Visual crowding is a fundamental limitation of object recognition in cluttered environments and is therefore highly relevant to everyday visual functioning. In daily life, objects are rarely viewed in isolation; instead, recognition often occurs in the presence of surrounding visual clutter, which can interfere with perception and impair performance on common tasks. Prior work has shown that crowding can adversely affect activities such as driving and face recognition, both of which are commonly reported as problematic by patients with glaucoma.^5^^,^^22^^,^^23^ We previously demonstrated that individuals with glaucoma exhibit greater visual crowding than healthy controls.^3^ The present study extends those findings by showing that worse crowding is also associated with poorer patient-reported QoL. To our knowledge, this study is the first to directly link a psychophysical measure of visual crowding to QoL in glaucoma.

In our cohort, each 1-degree increase in the better-eye global S_critical_ was associated with an approximately 5-point lower NEI VFQ-25 score, and this relationship remained significant after adjustment for age and visual field MD. This finding suggests that visual crowding captures aspects of visual dysfunction that are relevant to patients’ lived experience but are not fully reflected by SAP alone. Importantly, this association remained significant after additional adjustment for RNFL thickness, indicating that the relationship between visual crowding and QoL was not explained by structural measures of glaucomatous damage alone. One likely explanation is that SAP evaluates sensitivity to isolated, static light stimuli under highly controlled conditions, whereas many real-world visual tasks require the recognition of objects embedded in complex and cluttered scenes. As a result, SAP may incompletely capture forms of dysfunction that meaningfully affect QoL. This interpretation is consistent with prior work showing that conventional structural and functional measures explain only part of the variability in patient-reported outcomes in glaucoma. For example, Gracitelli et al.^20^ found that longitudinal changes in binocular RNFL thickness accounted for a substantial proportion of the change in NEI VFQ-25 scores, emphasizing that QoL in glaucoma is influenced by visual deficits that extend beyond standard perimetric measurements.

When considered separately by eye, a greater S_critical_ in both the better and worse eyes was associated with a poorer QoL in univariable analyses, but the association was stronger for the better eye. This pattern is consistent with prior evidence in glaucoma showing that binocular crowding is largely determined by the better eye and exhibits little binocular summation.^4^ More generally, previous work in normal vision has shown that crowding can attenuate binocular summation.^24^ It is also in line with prior studies showing that patient-reported QoL in glaucoma is more strongly related to visual function in the better eye than in the worse eye.^25^ In our study, the better-eye visual field MD likewise showed a stronger association with the NEI VFQ-25 score. Together, these findings suggest that dysfunction affecting the better eye may have a disproportionate impact on patients’ perceived visual ability and daily functioning.

The mechanisms underlying visual crowding are not yet fully understood. Several models have been proposed, including accounts based on feature integration, pooling or substitution of nearby features, midlevel perceptual grouping, and limitations of spatial attention.^26^ Although these frameworks differ in emphasis, they converge on the idea that crowding reflects the abnormal integration of visual information when nearby objects fall within an inappropriately large integration region.^27^ This idea is consistent with the well-known increase in crowding with retinal eccentricity, where larger integration fields in peripheral vision make object recognition more vulnerable to interference from nearby stimuli. In glaucoma, this framework may be particularly relevant because the structural and functional loss of retinal ganglion cells could alter the spatial scale over which visual information is integrated. Prior work has shown links between retinal ganglion cell sampling density and the extent of spatial integration,^28^ and experimental studies suggest that glaucomatous damage may be associated with the enlargement of functional integration areas.^27^^,^^29^^,^^30^ Such changes could provide a plausible basis for the increased crowding observed in glaucoma. Consistent with this hypothesis, our analysis demonstrated a progressive increase in the S_critical_ across glaucoma severity categories, with the highest values observed in eyes with advanced disease. These findings further support the hypothesis that visual crowding increases with glaucomatous damage.

This study has limitations. First, visual crowding was assessed using a psychophysical paradigm that is not yet standardized across studies, which may limit direct comparisons with prior reports using different testing methods. Second, testing was performed without eye tracking, so fixation instability could have influenced some measurements. Although fixation was monitored by an observer throughout the testing, we cannot exclude residual error from unrecognized fixation losses. Third, responses were given verbally and recorded by the examiner, which may have introduced a small degree of observer-related error, although this approach was chosen to reduce disruption of fixation and attention during testing. Fourth, we evaluated crowding monocularly rather than under binocular viewing conditions. Monocular testing allowed us to characterize the contribution of each eye separately, which is particularly relevant in glaucoma, given the frequent asymmetry of disease severity. Although binocular assessment may more closely reflect real-world visual experience, previous studies have suggested that binocular crowding in glaucoma is largely determined by the better eye and differs little from better-eye measurements.^4^ Consistent with these observations, the better-eye S_critical_ was the measure most strongly associated with vision-related QoL in our cohort. Therefore, although binocular testing may provide additional information, it is unlikely that it would have substantially altered the principal findings of the present study. Finally, although an individualized calibration procedure was performed before the crowding assessment to ensure adequate target visibility in each tested quadrant, target recognition was not reassessed during the subsequent testing phase, and the visibility of the flanking letters was not independently verified. The use of standardized Sloan letters and participant-specific letter size calibration was intended to minimize the influence of baseline letter recognition differences on crowding estimates. Nevertheless, we cannot completely exclude the possibility that residual limitations in letter visibility contributed to performance in some participants, independent of crowding effects. For example, localized glaucomatous visual field defects may have reduced the visibility of one or both flankers in some quadrants. Although completely invisible flankers would be expected to produce less interference with target identification, the effects of partially reduced or heterogeneous flanker visibility may be more complex. Therefore, residual limitations in flanker visibility could have influenced S_critical_ estimates in some quadrants, particularly in eyes with more advanced localized damage. Future studies incorporating microperimetry or a direct assessment of flanker visibility could further strengthen this methodology.

## Conclusions

Visual crowding was significantly associated with worse patient-reported QoL in glaucoma, particularly when present in the better eye. The persistence of this association after adjustment for age and visual field loss suggests that crowding reflects an aspect of visual dysfunction not fully captured by conventional perimetry. The assessment of visual crowding may, therefore, provide complementary information about the real-world functional consequences of glaucoma and help to identify patients who are more likely to experience vision-related disability.

## Supplementary Material

Supplement 1

Supplement 2

Supplement 3

## References

[1] Whitney D, Levi DM. Visual crowding: a fundamental limit on conscious perception and object recognition. *Trends Cogn Sci*. 2011; 15(4): 160–168. 10.1016/j.tics.2011.02.005 PMC3070834 21420894

[2] He Y, Legge GE. Linking crowding, visual span, and reading. *J Vis*. 2017; 17(11): 11. 10.1167/17.11.11 PMC5853154 28973564

[3] Ogata NG, Boer ER, Daga FB, et al. Visual crowding in glaucoma. *Invest Ophthalmol Vis Sci*. 2019; 60(2): 538–543. 10.1167/iovs.18-25150 PMC6361551 30716149

[4] Shamsi F, Liu R, Kwon M. Binocularly asymmetric crowding in glaucoma and a lack of binocular summation in crowding. *Invest Ophthalmol Vis Sci*. 2022; 63(1): 36. 10.1167/iovs.63.1.36 PMC8802025 35084432

[5] Stievenard A, Rouland JF, Peyrin C, et al. Sensitivity to central crowding for faces in patients with glaucoma. *J Glaucoma*. 2021; 30(2): 140–147. 10.1097/IJG.0000000000001710 33074958

[6] Tanriverdi D, Al-Nosairy KO, Hoffmann MB, Cornelissen FW. Assessing visual crowding in participants with preperimetric glaucoma using eye movement and manual response paradigms. *Transl Vis Sci Technol*. 2024; 13(9): 8. 10.1167/tvst.13.9.8 PMC11379081 39235398

[7] Lenoble Q, Lek JJ, McKendrick AM. Visual object categorisation in people with glaucoma. *Br J Ophthalmol*. 2016; 100(11): 1585–1590. 10.1136/bjophthalmol-2015-308289 27503395

[8] Glen FC, Smith ND, Crabb DP. Saccadic eye movements and face recognition performance in patients with central glaucomatous visual field defects. *Vision Res*. 2013; 82: 42–51. 10.1016/j.visres.2013.02.010 23485426

[9] Kwon M, Liu R, Patel BN, Girkin C. Slow reading in glaucoma: is it due to the shrinking visual span in central vision? *Invest Ophthalmol Vis Sci*. 2017; 58(13): 5810–5818. 10.1167/iovs.17-22560 PMC5808572 29131903

[10] Glen FC, Crabb DP, Smith ND, et al. Do patients with glaucoma have difficulty recognizing faces? *Invest Ophthalmol Vis Sci*. 2012; 53(7): 3629–3637. 10.1167/iovs.11-8538 22511628

[11] Smith ND, Crabb DP, Garway-Heath DF. An exploratory study of visual search performance in glaucoma. *Ophthalmic Physiol Opt*. 2011; 31: 225–232. 10.1111/j.1475-1313.2011.00836.x 21470272

[12] Haymes SA, LeBlanc RP, Nicolela MT, et al. Glaucoma and on-road driving performance. *Invest Ophthalmol Vis Sci*. 2008; 49(7): 3035–3041. 10.1167/iovs.07-1609 18326696

[13] Nassiri N, Mehravaran S, Nouri-Mahdavi K, Coleman AL. National Eye Institute Visual Function Questionnaire: usefulness in glaucoma. *Optom Vis Sci*. 2013; 90(8): 745–753. 10.1097/OPX.0000000000000003 23851305

[14] Keltner JL, Johnson CA, Quigg JM, et al. Confirmation of visual field abnormalities in the Ocular Hypertension Treatment Study. Ocular Hypertension Treatment Study Group. *Arch Ophthalmol*. 2000; 118(9): 1187–1194. 10.1001/archopht.118.9.1187 10980763

[15] Demirel S, De Moraes CG, Gardiner SK, et al. The rate of visual field change in the ocular hypertension treatment study. *Invest Ophthalmol Vis Sci*. 2012; 53(1): 224–227. 10.1167/iovs.10-7117 PMC3292359 22159015

[16] Berchuck SI, Mukherjee S, Medeiros FA. Estimating rates of progression and predicting future visual fields in glaucoma using a deep variational autoencoder. *Sci Rep*. 2019; 9(1): 18113. 10.1038/s41598-019-54653-6 PMC6888896 31792321

[17] Yohannan J, Wang J, Brown J, et al. Evidence-based criteria for assessment of visual field reliability. *Ophthalmology*. 2017; 124(11): 1612–1620. 10.1016/j.ophtha.2017.04.035 PMC5675138 28676280

[18] Song S, Levi DM, Pelli DG. A double dissociation of the acuity and crowding limits to letter identification, and the promise of improved visual screening. *J Vis*. 2014; 14(5): 3. 10.1167/14.5.3 PMC4021854 24799622

[19] Mangione CM, Lee PP, Gutierrez PR, et al. Development of the 25-item National Eye Institute Visual Function Questionnaire. *Arch Ophthalmol*. 2001; 119(7): 1050–1058. 10.1001/archopht.119.7.1050 11448327

[20] Gracitelli CP, Abe RY, Tatham AJ, et al. Association between progressive retinal nerve fiber layer loss and longitudinal change in quality of life in glaucoma. *JAMA Ophthalmol*. 2015; 133(4): 384–390. 10.1001/jamaophthalmol.2014.5319 PMC4533995 25569808

[21] Andrich D . Rating scales and Rasch measurement. *Expert Rev Pharmacoecon Outcomes Res*. 2011; 11(5): 571–585. 10.1586/erp.11.59 21958102

[22] Xia Y, Manassi M, Nakayama K, et al. Visual crowding in driving. *J Vis*. 2020; 20(6): 1. 10.1167/jov.20.6.1 PMC7416909 32492098

[23] Farzin F, Rivera SM, Whitney D. Holistic crowding of Mooney faces. *J Vis*. 2009; 9(6):18: 1–5. 10.1167/9.6.18 PMC2857385 19761309

[24] Siman-Tov Z, Lev M, Polat U. Binocular summation is affected by crowding and tagging. *Sci Rep*. 2021; 11(1): 4843. 10.1038/s41598-021-83510-8 PMC7921124 33649371

[25] van Gestel A, Webers CA, Beckers HJ, et al. The relationship between visual field loss in glaucoma and health-related quality-of-life. *Eye (Lond)*. 2010; 24(12): 1759–1769. 10.1038/eye.2010.133 21057519

[26] He S, Cavanagh P, Intriligator J. Attentional resolution and the locus of visual awareness. *Nature*. 1996; 383(6598): 334–337. 10.1038/383334a0 8848045

[27] Redmond T, Garway-Heath DF, Zlatkova MB, Anderson RS. Sensitivity loss in early glaucoma can be mapped to an enlargement of the area of complete spatial summation. *Invest Ophthalmol Vis Sci*. 2010; 51(12): 6540–6548. 10.1167/iovs.10-5718 20671278

[28] Kwon M, Liu R. Linkage between retinal ganglion cell density and the nonuniform spatial integration across the visual field. *Proc Natl Acad Sci USA*. 2019; 116(9): 3827–3836. 10.1073/pnas.1817076116 PMC6397585 30737290

[29] King WM, Sarup V, Sauve Y, et al. Expansion of visual receptive fields in experimental glaucoma. *Vis Neurosci*. 2006; 23(1): 137–142. 10.1017/S0952523806231122 16597357

[30] Ahmed FA, Chaudhary P, Sharma SC. Effects of increased intraocular pressure on rat retinal ganglion cells. *Int J Dev Neurosci*. 2001; 19(2): 209–218. 10.1016/s0736-5748(00)00073-3 11255034

