# Supplementary material for: Effect of Visual Crowding on Quality of Life in Glaucoma Patients
Source: Invest Ophthalmol Vis Sci. 2026 Oct 7;67(12):13. doi: 10.1167/iovs.67.12.13 (PMC13657127; doi:10.1167/iovs.67.12.13)
Supplement: Supplementary file 1 [file iovs-67-12-13_s001.docx]

**Supplementary Table** **S1**. Multivariable models assessing the effect of crowding critical spacing (S_critical_) in the better and worse eyes on Rasch scores from the National Eye Institute Visual Function Questionnaire (NEI VFQ-25), with the addition of retinal nerve fiber layer (RNFL) thickness as potential predictor.

|  | **Multivariable Model Better Eye** |  | **Multivariable Model Worse Eye** |  |
| --- | --- | --- | --- | --- |
|  |  |  |  |  |
|  | Effect on NEI VFQ-25 |  | Effect on NEI VFQ-25 |  |
|  | (Rasch score) |  | (Rasch score) |  |
| **Characteristic** | **Coefficient (95% CI)** | **P Value** | **Coefficient (95% CI)** | **P Value** |
| Age, per decade older | 2.91 (−2.44 to 8.28) | 0.286 | 1.02 (−4.06 to 6.11) | 0.694 |
| S_critical_, per degree greater | −5.19 (−9.74 to −0.63) | **0.025** | −1.08 (−3.32 to 1.14) | 0.339 |
| SAP 24-2 MD, per dB lower | −0.78 (−1.56 to −0.01) | **0.048** | −0.83 (−1.86 to 0.19) | 0.114 |
| RNFL, per 10 µm lower | 1.03 (−2.78 to 4.85) | 0.594 | 1.53 (−2.23 to 5.31) | 0.426 |

Boldface indicates statistical significance (P < 0.05).

**Abbreviations:** CI = confidence interval; dB = decibel; MD = mean deviation; NEI VFQ = National Eye Institute Visual Function Questionnaire; RNFL = retinal nerve fiber layer; S_critical_ = critical spacing.

**Supplementary Table** **S2**. Multivariable models assessing the effect of worst quadrant crowding critical spacing (S_critical_) in the better and worse eyes on Rasch scores from the National Eye Institute Visual Function Questionnaire (NEI VFQ-25).

|  | **Multivariable Model Better Eye** |  | **Multivariable Model Worse Eye** |  |
| --- | --- | --- | --- | --- |
|  |  |  |  |  |
|  | Effect on NEI VFQ-25 |  | Effect on NEI VFQ-25 |  |
|  | (Rasch score) |  | (Rasch score) |  |
| **Characteristic** | **Coefficient (95% CI)** | **P Value** | **Coefficient (95% CI)** | **P Value** |
| Age, per decade older | 4.17 (−0.31 to 8.65) | 0.068 | 1.13 (−3.89 to 6.16) | 0.659 |
| Worst quadrant S_critical_, per degree greater | −5.31 (−9.40 to −1.21) | **0.011** | −0.87 (−2.29 to 0.54) | 0.228 |
| SAP 24-2 MD, per dB lower | −0.61 (−1.21 to −0.02) | **0.044** | −0.56 (−1.41 to 0.28) | 0.193 |

Boldface indicates statistical significance (P < 0.05).

**Abbreviations:** CI = confidence interval; dB = decibel; MD = mean deviation; NEI VFQ = National Eye Institute Visual Function Questionnaire; S_critical_ = critical spacing.
