# Supplementary material for: Effect of Visual Crowding on Quality of Life in Glaucoma Patients
Source: Invest Ophthalmol Vis Sci. 2026 Oct 7;67(12):13. doi: 10.1167/iovs.67.12.13 (PMC13657127; doi:10.1167/iovs.67.12.13)
Supplement: Supplementary file 2 [file iovs-67-12-13_s002.docx]

**Supplementary Appendix S1**

Manuscript #: IOVS-26-46770R2

Manuscript Title: "Effect of Visual Crowding on Quality of Life in Glaucoma Patients"

**Supplementary Appendix S1. Post Hoc QUEST Robustness Simulation**

# Purpose

A post hoc Monte Carlo simulation was performed to further evaluate whether a 20-trial adaptive procedure would be expected to recover thresholds in the critical spacing (S_critical_) range observed in the cohort. Specifically, we tested the fixed nominal QUEST starting value used in the implementation, 0.3°, by simulating 20-trial staircases initialized at 0.3° across the observed S_critical_ values. As a complementary stress test, we also evaluated additional starting-value offsets ranging from 0.5× to 2.0× the true threshold.

Because our final S_critical_ estimate was obtained from a logistic fit to trial-level correct/incorrect responses, rather than from participant-specific QUEST posterior SD, reliability was quantified using simulation-derived variability of the recovered thresholds, including SD, coefficient of variation (CV), and 5th to 95th percentile ranges. The simulation was intended as a robustness check of expected QUEST-like behavior under the stated task and psychometric assumptions, not as a re-estimation of participant-specific S_critical_ values from the original testing sessions.

# Task Parameters Used in the Simulation

| **Parameter** | **Value** |
| --- | --- |
| Task design | 9-alternative forced-choice letter-identification task |
| Theoretical chance performance | 1/9 = 11.1% |
| Threshold criterion | 75% correct performance |
| Number of trials | 20 trials per eligible quadrant |
| Threshold outcome | S_critical_, in degrees of visual angle |

S_critical_ = critical spacing.

# Implementation-Informed Simulation Assumptions

| **Assumption** | **Primary value** |
| --- | --- |
| Psychometric function | Weibull, in log10 target-flanker spacing |
| Guess rate, gamma | 1/9 |
| Threshold criterion | 75% correct |
| Psychometric slope, beta | 3.0 |
| Lapse rate, delta | 0.01 |
| Prior SD | 2.0 log10 units |
| Fixed nominal starting value | 0.3° |
| Illustrative allowable spacing range | 0.2° to 15.0° |
| Trial placement | Posterior mode, clipped to the illustrative allowable range |
| Final simulated estimate | Posterior median after 20 trials |
| Monte Carlo repetitions | 3,000 per primary condition |

The lower bound of 0.2 degrees was selected so that the fixed nominal starting value of 0.3 degrees is included within the simulated allowable range and is not artificially clipped at the first trial. The upper bound of 15.0 degrees was selected as a broad interval relative to the observed group-level S_critical_ values. The primary reliability metrics were bias, SD, coefficient of variation, and 5th to 95th percentile ranges of the recovered thresholds. Boundary-clipping rates were retained as diagnostic outputs of the simulated staircase behavior and were not interpreted as participant-level reliability estimates.

# Simulation Design

The primary fixed-start analysis used simulated true S_critical_ values spanning the observed mild, moderate, and severe glaucoma group means: 4.14°, 4.89°, and 6.42°, respectively. For each of these three thresholds, 3,000 independent 20-trial staircases were simulated with the fixed nominal QUEST starting value of 0.3°.

As a complementary stress test, the starting value was also varied across deliberately conservative offsets, from 0.5× to 2.0× the true threshold. This stress test was performed for each of the three simulated true thresholds, with 3,000 independent 20-trial staircases per condition.

A sensitivity analysis varied the assumed psychometric slope and lapse rate to confirm that the qualitative conclusion was not dependent on a single psychometric-function assumption.

# Outcomes

Simulation outputs included mean estimated threshold, percent bias relative to the simulated true threshold, SD of the 20-trial threshold estimate, coefficient of variation, median absolute error, 5th to 95th percentile range, and boundary-clipping diagnostics.

# Results

In the fixed nominal 0.3° starting-value analysis, each true-threshold condition was simulated using 3,000 independent 20-trial staircases. When initialized at this fixed nominal starting value, the procedure recovered thresholds across the observed S_critical_ range with a mean absolute bias of 0.54%, an SD range of approximately 0.62° to 0.92°, and a mean CV of 15.0%.

| **True** S_critical_ | **Start** | **Mean recovered** | **Bias** | **SD** | **CV** | **5th-95th percentile range** |
| --- | --- | --- | --- | --- | --- | --- |
| 4.14° | 0.3° | 4.163° | +0.54% | 0.622° | 15.0% | 3.260° to 5.112° |
| 4.89° | 0.3° | 4.933° | +0.88% | 0.769° | 15.7% | 3.842° to 6.067° |
| 6.42° | 0.3° | 6.433° | +0.20% | 0.921° | 14.4% | 5.037° to 7.957° |

In the complementary starting-value offset stress test, recovered threshold estimates showed minimal sensitivity to starting-value offsets from 0.5× to 2.0× the true threshold. Across mis-anchored starting-value conditions, the mean absolute bias was 0.29%. The SD of a single 20-trial estimate ranged from approximately 0.53° to 0.84°, corresponding to a mean CV of 12.9%.

Sensitivity analyses varying the assumed psychometric slope and lapse rate produced the same qualitative conclusion. The adaptive procedure remained generally robust to substantial starting-value offsets, although variability increased under more unfavorable simulated psychometric assumptions.

# Interpretation

These simulations provide supportive evidence that a 20-trial QUEST-like procedure, configured with the stated task parameters and implementation-informed assumptions, would be expected to recover thresholds in the observed S_critical_ range. In particular, the fixed nominal starting value of 0.3° did not prevent recovery of thresholds in the 4-6° range under the simulated assumptions.

The simulation should be interpreted as a post hoc robustness check of the expected behavior of the adaptive procedure, rather than a re-estimation of participant-specific S_critical_ values from the original testing sessions.

# Supplementary Code

The Python code used to perform this post hoc Monte Carlo simulation is provided as Supplementary Code S1.
