# Supplementary material for: Effect of Visual Crowding on Quality of Life in Glaucoma Patients
Source: Invest Ophthalmol Vis Sci. 2026 Oct 7;67(12):13. doi: 10.1167/iovs.67.12.13 (PMC13657127; doi:10.1167/iovs.67.12.13)
Supplement: Supplementary file 3 [file iovs-67-12-13_s003.docx]

**Supplementary Code S1**

Manuscript #: IOVS-26-46770R2

Manuscript Title: "Effect of Visual Crowding on Quality of Life in Glaucoma Patients"

```
"""
Manuscript #: IOVS-26-46770R2
Manuscript Title: "Effect of Visual Crowding on Quality of Life in Glaucoma Patients"

Supplementary Code S1. QUEST Robustness Simulation

This script performs a post hoc Monte Carlo simulation of a QUEST-like adaptive
staircase for estimating crowding critical spacing (Scritical).

Purpose
-------
The simulation was designed to further evaluate whether a 20-trial adaptive
procedure would be expected to recover thresholds in the critical spacing (Scritical) range
observed in the cohort. Specifically, we tested the fixed nominal QUEST starting
value used in the implementation, 0.3°, by simulating 20-trial staircases
initialized at 0.3° across the observed Scritical values. As a complementary stress test,
we also evaluated additional starting-value offsets ranging from 0.5× to 2.0× the true threshold.

Because our final Scritical estimate was obtained from a logistic fit to
trial-level correct/incorrect responses, rather than from participant-specific
QUEST posterior SD, reliability is quantified using simulation-derived
variability of the recovered thresholds, including SD, coefficient of
variation, and 5th to 95th percentile ranges.

Manuscript-consistent task structure
------------------------------------
    - 9-alternative forced-choice letter-identification task
    - chance performance = 1/9 = 11.1%
    - threshold criterion = 75% correct
    - 20 trials per eligible quadrant

Implementation-informed simulation assumptions
----------------------------------------------
    - Weibull psychometric function in log10 target-flanker spacing
    - beta = 3.0 in the primary analysis
    - lapse rate delta = 0.01 in the primary analysis
    - broad prior SD = 2.0 log10 units
    - fixed nominal QUEST starting value = 0.3 degrees for the fixed-start test
    - illustrative allowable spacing range = 0.2 to 15.0 degrees

The lower bound of 0.2 degrees was selected so that the fixed nominal starting
value of 0.3 degrees is included within the simulated allowable range and is not
artificially clipped at the first trial. The upper bound of 15.0 degrees was
selected as a broad interval relative to the observed group-level Scritical
values. The primary reliability metrics were bias, SD, coefficient of variation,
and 5th to 95th percentile ranges of the recovered thresholds. Boundary-clipping
rates were retained as diagnostic outputs of the simulated staircase behavior
and were not interpreted as participant-level reliability estimates.

Simulation design
-----------------
The simulation has three parts:

1. Fixed nominal starting-value analysis:
   Simulated true Scritical values were set to the observed mild, moderate, and
   severe glaucoma group means: 4.14, 4.89, and 6.42 degrees. All simulated
   staircases were initialized at 0.3 degrees to directly test the fixed nominal
   QUEST starting value identified from the implementation.

2. Starting-value offset stress test:
   For each true threshold, the starting value was varied from 0.5x to 2.0x
   the true threshold to evaluate robustness to deliberately unfavorable
   starting-value offsets.

3. Sensitivity analysis:
   The psychometric slope and lapse rate were varied to confirm that the
   qualitative conclusion was not dependent on a single psychometric-function
   assumption.

Simulation outcomes
-------------
    - mean estimated threshold
    - percent bias relative to the simulated true threshold
    - SD of the 20-trial estimate
    - coefficient of variation
    - median absolute error
    - 5th to 95th percentile interval
    - boundary-clipping diagnostics, retained as diagnostic outputs only

Interpretation
-------------
These simulations provide supportive evidence that a 20-trial QUEST-like procedure,
configured with the stated task parameters and implementation-informed assumptions,
would be expected to recover thresholds in the observed Scritical range.
In particular, the fixed nominal starting value of 0.3° did not prevent recovery of
thresholds in the 4-6° range under the simulated assumptions. The simulation should be
interpreted as a post hoc robustness check of the expected behavior of the adaptive procedure,
rather than a re-estimation of participant-specific Scritical values from the original testing sessions.

Run
---
    python Supplementary_Code_S1_IOVS-26-46770R2_QUEST_Robustness_Simulation.py

Optional quick test, not for reporting final results
----------------------------------------------------
    N_SIMS=100 SENS_N_SIMS=100 python Supplementary_Code_S1_IOVS-26-46770R2_QUEST_Robustness_Simulation.py
"""

from __future__ import annotations

import os
from typing import Dict, List, Tuple

import numpy as np


# ----------------------------------------------------------------------
# Manuscript-consistent task parameters
# ----------------------------------------------------------------------
GAMMA = 1.0 / 9.0      # 9-AFC theoretical guess rate
P_THRESH = 0.75        # Scritical criterion: 75% correct
N_TRIALS = 20          # trials per eligible quadrant


# ----------------------------------------------------------------------
# Primary implementation-informed psychometric assumptions
# ----------------------------------------------------------------------
DEFAULT_BETA = 3.0     # Weibull psychometric slope
DEFAULT_DELTA = 0.01   # lapse rate
START_SD = 2.0         # broad prior width in log10 units


# ----------------------------------------------------------------------
# Simulated allowable spacing range
# ----------------------------------------------------------------------
ALLOWABLE_MIN_DEG = 0.2
ALLOWABLE_MAX_DEG = 15.0
LOG_MIN = np.log10(ALLOWABLE_MIN_DEG)
LOG_MAX = np.log10(ALLOWABLE_MAX_DEG)

# The posterior grid is wider than the allowable presentation range. This lets
# the simulation detect when the staircase would suggest a value outside the
# allowable range before that value is clipped for presentation.
GRID_POINTS = int(os.environ.get("GRID_POINTS", "800"))
GRID = np.linspace(np.log10(0.2), np.log10(60.0), GRID_POINTS)

# Default Monte Carlo repetitions. Environment variables allow quick checks.
N_SIMS = int(os.environ.get("N_SIMS", "3000"))
SENS_N_SIMS = int(os.environ.get("SENS_N_SIMS", "1000"))


def compute_threshold_offset(
    beta: float,
    gamma: float,
    delta: float,
    p_thresh: float = P_THRESH,
) -> float:
    """
    Compute the log10 offset that makes the simulated true threshold correspond
    exactly to the desired percent-correct criterion.

    Psychometric function:
        P(correct) = gamma + (1 - gamma - delta) *
                     (1 - exp(-10 ** (beta * ((x - t) + k))))

    where x is presented log10 spacing, t is true log10 threshold, beta is the
    psychometric slope, gamma is the guess rate, delta is the lapse rate, and k
    is the offset that anchors t to the desired p_thresh.
    """
    a = (p_thresh - gamma) / (1.0 - gamma - delta)
    if not (0.0 < a < 1.0):
        raise ValueError("Invalid gamma, delta, or p_thresh combination.")
    return float(np.log10(-np.log(1.0 - a)) / beta)


def weibull_p_correct(
    x_log10: np.ndarray | float,
    t_log10: np.ndarray | float,
    beta: float,
    k: float,
    gamma: float = GAMMA,
    delta: float = DEFAULT_DELTA,
) -> np.ndarray | float:
    """Return expected percent correct for a Weibull function in log10 spacing."""
    diff = (x_log10 - t_log10) + k
    return gamma + (1.0 - gamma - delta) * (1.0 - np.exp(-10.0 ** (beta * diff)))


def run_quest_condition(
    true_threshold_deg: float,
    start_deg: float,
    beta: float,
    delta: float,
    rng: np.random.Generator,
    n_sims: int = N_SIMS,
) -> Tuple[np.ndarray, np.ndarray, np.ndarray]:
    """
    Simulate n_sims independent 20-trial QUEST-like staircases for one
    threshold/start/beta/delta condition.

    Trial placement:
        posterior mode, clipped to the illustrative allowable range.

    Final simulated estimate:
        posterior median after 20 trials.
    """
    k = compute_threshold_offset(beta, GAMMA, delta)
    t_true = np.log10(true_threshold_deg)
    start = np.log10(start_deg)

    log_prior = -0.5 * ((GRID - start) / START_SD) ** 2
    log_prior -= np.max(log_prior)
    prior = np.exp(log_prior)
    prior /= prior.sum()

    posterior = np.tile(prior, (n_sims, 1))
    hit_lower = np.zeros(n_sims, dtype=bool)
    hit_upper = np.zeros(n_sims, dtype=bool)

    for _ in range(N_TRIALS):
        mode_idx = np.argmax(posterior, axis=1)
        x_suggested = GRID[mode_idx]

        hit_lower |= x_suggested < LOG_MIN
        hit_upper |= x_suggested > LOG_MAX

        x_next = np.clip(x_suggested, LOG_MIN, LOG_MAX)

        p_true = weibull_p_correct(x_next, t_true, beta, k, delta=delta)
        correct = rng.random(n_sims) < p_true

        p_model = weibull_p_correct(
            x_next[:, None],
            GRID[None, :],
            beta,
            k,
            delta=delta,
        )
        p_model = np.clip(p_model, 1e-9, 1.0 - 1e-9)
        likelihood = np.where(correct[:, None], p_model, 1.0 - p_model)

        posterior *= likelihood
        posterior /= posterior.sum(axis=1, keepdims=True)

    cdf = np.cumsum(posterior, axis=1)
    median_idx = np.argmax(cdf >= 0.5, axis=1)
    estimates_deg = 10.0 ** GRID[median_idx]

    return estimates_deg, hit_lower, hit_upper


def summarize(
    estimates: np.ndarray,
    hits_lower: np.ndarray,
    hits_upper: np.ndarray,
    true_threshold_deg: float,
) -> Dict[str, float]:
    """Compute threshold-recovery and variability metrics for one condition."""
    estimates = np.asarray(estimates, dtype=float)
    hits_lower = np.asarray(hits_lower, dtype=bool)
    hits_upper = np.asarray(hits_upper, dtype=bool)

    bias_pct = 100.0 * (estimates.mean() - true_threshold_deg) / true_threshold_deg
    sd = estimates.std(ddof=0)
    cv_pct = 100.0 * sd / true_threshold_deg
    mae = np.median(np.abs(estimates - true_threshold_deg))
    p5, p95 = np.percentile(estimates, [5, 95])
    lower_rate = 100.0 * hits_lower.mean()
    upper_rate = 100.0 * hits_upper.mean()
    interior_rate = 100.0 * (~hits_lower & ~hits_upper).mean()

    return {
        "mean_est": float(estimates.mean()),
        "bias_pct": float(bias_pct),
        "sd": float(sd),
        "cv_pct": float(cv_pct),
        "mae": float(mae),
        "p5": float(p5),
        "p95": float(p95),
        "lower_rate": float(lower_rate),
        "upper_rate": float(upper_rate),
        "interior_rate": float(interior_rate),
    }


def print_condition_row(label: str, offset: float, start_deg: float, stats: Dict[str, float]) -> None:
    """Print one formatted condition row."""
    print(
        f"{label:<20}"
        f"{offset:<8.2f}"
        f"{start_deg:<8.2f}"
        f"{stats['mean_est']:<9.3f}"
        f"{stats['bias_pct']:<8.2f}"
        f"{stats['sd']:<7.3f}"
        f"{stats['cv_pct']:<6.1f}"
        f"{stats['mae']:<7.3f}"
        f"{stats['p5']:<7.3f}"
        f"{stats['p95']:<7.3f}"
        f"{stats['lower_rate']:<9.1f}"
        f"{stats['upper_rate']:<9.1f}"
        f"{stats['interior_rate']:<10.1f}"
    )


def starting_value_robustness_analysis() -> List[Tuple[str, float, Dict[str, float]]]:
    """Analyze stress-test starts ranging from 0.5x to 2.0x true threshold."""
    rng = np.random.default_rng(42)
    true_thresholds = {
        "mild (4.14 deg)": 4.14,
        "moderate (4.89 deg)": 4.89,
        "severe (6.42 deg)": 6.42,
    }
    offsets = [0.50, 0.75, 1.00, 1.50, 2.00]

    print("=" * 132)
    print("ANALYSIS 2: Starting-value offset stress test")
    print(
        f"(N_TRIALS={N_TRIALS}, N_SIMS={N_SIMS}, beta={DEFAULT_BETA}, "
        f"delta={DEFAULT_DELTA}, gamma={GAMMA:.4f}, pThreshold={P_THRESH}, "
        f"allowable range=[{ALLOWABLE_MIN_DEG},{ALLOWABLE_MAX_DEG}] deg)"
    )
    print("=" * 132)

    header = (
        f"{'True thr.':<20}{'Offset':<8}{'Start':<8}{'MeanEst':<9}{'Bias%':<8}"
        f"{'SD':<7}{'CV%':<6}{'MAE':<7}{'P5':<7}{'P95':<7}"
        f"{'Lo-hit%':<9}{'Hi-hit%':<9}{'Interior%':<10}"
    )
    print(header)
    print("-" * 132)

    results = []
    for label, true_threshold in true_thresholds.items():
        for offset in offsets:
            start_deg = true_threshold * offset
            estimates, lower_hits, upper_hits = run_quest_condition(
                true_threshold,
                start_deg,
                DEFAULT_BETA,
                DEFAULT_DELTA,
                rng,
            )
            stats = summarize(estimates, lower_hits, upper_hits, true_threshold)
            results.append((label, offset, stats))
            print_condition_row(label, offset, start_deg, stats)

    print("-" * 132)
    mis_anchored = [stats for (_, offset, stats) in results if offset != 1.00]
    mean_abs_bias = np.mean([abs(stats["bias_pct"]) for stats in mis_anchored])
    mean_cv = np.mean([stats["cv_pct"] for (_, _, stats) in results])
    mean_interior = np.mean([stats["interior_rate"] for (_, _, stats) in results])

    print(f"Mean |bias| across mis-anchored conditions:       {mean_abs_bias:.2f}%")
    print(f"Mean CV across all conditions:                    {mean_cv:.1f}%")
    print(f"Mean % of staircases never touching a boundary:   {mean_interior:.1f}%")
    print()

    return results


def fixed_nominal_start_analysis() -> List[Tuple[str, float, Dict[str, float]]]:
    """Analyze the fixed nominal 0.3-degree starting value for all thresholds."""
    rng = np.random.default_rng(777)
    true_thresholds = {
        "mild (4.14 deg)": 4.14,
        "moderate (4.89 deg)": 4.89,
        "severe (6.42 deg)": 6.42,
    }
    fixed_start_deg = 0.3

    print("=" * 132)
    print("ANALYSIS 1: Fixed nominal starting value analysis")
    print(
        f"(N_TRIALS={N_TRIALS}, N_SIMS={N_SIMS}, fixed start={fixed_start_deg} deg, "
        f"beta={DEFAULT_BETA}, delta={DEFAULT_DELTA}, gamma={GAMMA:.4f}, "
        f"pThreshold={P_THRESH}, allowable range=[{ALLOWABLE_MIN_DEG},{ALLOWABLE_MAX_DEG}] deg)"
    )
    print("=" * 132)

    header = (
        f"{'True thr.':<20}{'Offset':<8}{'Start':<8}{'MeanEst':<9}{'Bias%':<8}"
        f"{'SD':<7}{'CV%':<6}{'MAE':<7}{'P5':<7}{'P95':<7}"
        f"{'Lo-hit%':<9}{'Hi-hit%':<9}{'Interior%':<10}"
    )
    print(header)
    print("-" * 132)

    results = []
    for label, true_threshold in true_thresholds.items():
        offset = fixed_start_deg / true_threshold
        estimates, lower_hits, upper_hits = run_quest_condition(
            true_threshold,
            fixed_start_deg,
            DEFAULT_BETA,
            DEFAULT_DELTA,
            rng,
        )
        stats = summarize(estimates, lower_hits, upper_hits, true_threshold)
        results.append((label, offset, stats))
        print_condition_row(label, offset, fixed_start_deg, stats)

    print("-" * 132)
    mean_abs_bias = np.mean([abs(stats["bias_pct"]) for (_, _, stats) in results])
    mean_cv = np.mean([stats["cv_pct"] for (_, _, stats) in results])
    mean_interior = np.mean([stats["interior_rate"] for (_, _, stats) in results])

    print(f"Mean |bias| across fixed-0.3-deg conditions:   {mean_abs_bias:.2f}%")
    print(f"Mean CV across fixed-0.3-deg conditions:       {mean_cv:.1f}%")
    print(f"Mean % of staircases never touching a boundary:{mean_interior:.1f}%")
    print()

    return results


def beta_delta_sensitivity_analysis() -> List[Tuple[float, float, float, Dict[str, float]]]:
    """Analyze whether conclusions are robust to alternate beta and delta values."""
    rng = np.random.default_rng(123)
    true_threshold = 4.89  # moderate glaucoma group mean
    offsets = [0.50, 2.00]
    beta_values = [2.0, 3.0, 5.0]
    delta_values = [0.01, 0.02, 0.05]

    print("=" * 100)
    print("ANALYSIS 3: Sensitivity to assumed beta (slope) and delta (lapse rate)")
    print(
        f"(true threshold={true_threshold} deg, N_TRIALS={N_TRIALS}, "
        f"SENS_N_SIMS={SENS_N_SIMS}, pThreshold={P_THRESH})"
    )
    print("=" * 100)
    print(
        f"{'beta':<7}{'delta':<8}{'Offset':<8}{'MeanEst':<9}"
        f"{'Bias%':<8}{'SD':<7}{'CV%':<6}{'Lo-hit%':<9}{'Hi-hit%':<9}"
    )
    print("-" * 82)

    sensitivity_results = []
    for beta in beta_values:
        for delta in delta_values:
            for offset in offsets:
                start_deg = true_threshold * offset
                estimates, lower_hits, upper_hits = run_quest_condition(
                    true_threshold,
                    start_deg,
                    beta,
                    delta,
                    rng,
                    n_sims=SENS_N_SIMS,
                )
                stats = summarize(estimates, lower_hits, upper_hits, true_threshold)
                sensitivity_results.append((beta, delta, offset, stats))
                print(
                    f"{beta:<7.1f}"
                    f"{delta:<8.2f}"
                    f"{offset:<8.2f}"
                    f"{stats['mean_est']:<9.3f}"
                    f"{stats['bias_pct']:<8.2f}"
                    f"{stats['sd']:<7.3f}"
                    f"{stats['cv_pct']:<6.1f}"
                    f"{stats['lower_rate']:<9.1f}"
                    f"{stats['upper_rate']:<9.1f}"
                )

    print()
    return sensitivity_results


if __name__ == "__main__":
    fixed_nominal_start_analysis()
    starting_value_robustness_analysis()
    beta_delta_sensitivity_analysis()
```
